# Formation of Supported Lipid Bilayers Derived from Vesicles of Various Compositional Complexity on Conducting Polymer/Silica Substrates

**DOI:** 10.1021/acs.langmuir.1c00175

**Published:** 2021-04-30

**Authors:** Hanna Ulmefors, Josefin Nissa, Hudson Pace, Olov Wahlsten, Anders Gunnarsson, Daniel T. Simon, Magnus Berggren, Fredrik Höök

**Affiliations:** † Division of Nano and Biological Physics, Department of Physics, Chalmers University of Technology, 412 96 Gothenburg, Sweden; ‡ Laboratory of Organic Electronics, Department of Science and Technology, Linköping University, 601 74 Norrköping, Sweden; § Discovery Sciences, BioPharmaceuticals R&D, AstraZeneca, Pepparedsleden 1, 431 83 Mölndal, Sweden

## Abstract

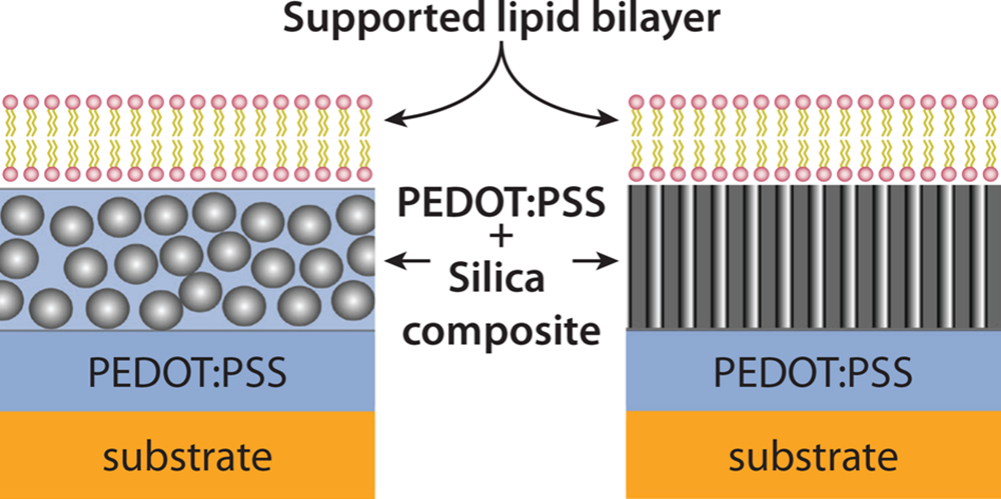

Supported lipid bilayers (SLBs) serve important roles as minimalistic models of cellular membranes in multiple diagnostic and pharmaceutical applications as well as in the strive to gain fundamental insights about their complex biological function. To further expand the utility of SLBs, there is a need to go beyond simple lipid compositions to thereby better mimic the complexity of native cell membranes, while simultaneously retaining their compatibility with a versatile range of analytical platforms. To meet this demand, we have in this work explored SLB formation on PEDOT:PSS/silica nanoparticle composite films and mesoporous silica films, both capable of transporting ions to an underlying conducting PEDOT:PSS film. The SLB formation process was evaluated by using the quartz crystal microbalance with dissipation (QCM-D) monitoring, total internal reflection fluorescence (TIRF) microscopy, and fluorescence recovery after photobleaching (FRAP) for membranes made of pure synthetic lipids with or without the reconstituted membrane protein β-secretase 1 (BACE1) as well as cell-derived native lipid vesicles containing overexpressed BACE1. The mesoporous silica thin film was superior to the PEDOT:PSS/silica nanoparticle composite, providing successful formation of bilayers with high lateral mobility and low defect density even for the most complex native cell membranes.

## Introduction

The cellular plasma membrane, composed of lipids, cholesterol, glycans, and proteins, encapsulates the cytosolic components and serves as a selectively permeable barrier. Membrane proteins fulfill vital functions as signal transducers, motors, anchors, and selective transporters of ions across the cell membrane and their receptors are a central class of pharmaceutical and diagnostic targets.^1−3^ To maintain their native structure and function, the proteins need to reside in their natural environment of the fluid lipid bilayer.

Supported lipid bilayers (SLBs) formed on planar surfaces, first demonstrated by Tamm et al.,^4^ are today widely used as simplified models of the cell membrane. These supported bilayers provide a robust platform and the possibility to increase the compositional complexity has increased over the years, now ranging from bilayers containing phospholipids with just a few additional components to structures derived from native membranes.^5−7^ They are compatible with a range of surface-based analytical tools, such as quartz crystal microbalance with dissipation (QCM-D) monitoring,^8−10^ total internal reflection fluorescence microscopy (TIRFM),^11,12^ and atomic force microscopy (AFM).^13,14^ Traditionally, SLBs are formed on hydrophilic and smooth surfaces, like glass^15^ or mica,^13^ due to the ability of these materials to facilitate spontaneous bilayer formation through vesicle adsorption and subsequent fusion.

Conducting polymers are organic-based semiconductors used in a variety of applications, including the increasingly ubiquitous organic electrochemical transistors (OECT). OECTs are incorporated in various bioelectronic devices for biomedical research and are attractive due to their biocompatibility, capacity to efficiently transduce ionic currents into electronic signals and act as amplifiers, and the ability to be integrated with a large variety of substrates.^15−17^ By introduction of a conducting polymer to the underlying substrate of a supported lipid bilayer, it is possible to develop label-free biosensor platforms that can directly monitor the integrity of the resistive bilayer, protein binding events, and ion channel transport.^18,19^ Owens and co-workers recently showed formation of lipid bilayers derived from synthetic lipid vesicles (mixtures of diphytanoylphospatidylcholine, DPhPC, and diphytanoylphosphoethanolamine, DPhPE), on the conducting polymer poly(3,4-ethylenedioxythiophene)–polystyrenesulfonate (PEDOT:PSS, one of the most commonly used materials in organic bioelectronics).^18^ These bilayers, however, were incomplete, with a large amount of unruptured vesicles remaining on the surface after vesicle fusion. Zhang et al. managed to improve the quality of the DPhPC bilayer to some extent by increasing the substrate polarity with ethylene glycol incorporated in the PEDOT:PSS film.^19^ The typical hydrophobic character of these polymers and a rough surface structure are not ideal conditions for bilayer formation. Thus, the challenge of obtaining defect-free bilayers with highly mobile lipids on bioelectronic substrates still remains. Moreover, DPhPC and DPhPE are synthetic lipids and do not match the complexity of eukaryotic or bacterial membranes.^20^

In this work, thin films chosen for their vesicle fusion capabilities were spin-coated on top of pure PEDOT:PSS and assessed for their suitability as substrates for bilayer formation. The two resulting substrates have the potential to transport ions through the thin film down to the underlying conducting PEDOT:PSS film. This approach allows for utilization of the attractive features of the pure conducting polymer film without being in direct contact with the bilayer. Inspired by the compatibility of silica as a suitable substrate for bilayer formation, both platforms include different forms of silica structures. In the first approach, a composite thin film (CTF) composed of a network of silica nanoparticles and PEDOT:PSS was spin-coated on top of the pure PEDOT:PSS film. The silica/PEDOT:PSS ratio was varied to determine optimal conditions for bilayer formation. In the second approach, a mesoporous silica thin film (MPTF) was synthesized on top of the PEDOT:PSS layer, with an open pore structure through the thin film. The two different platforms were investigated to find the most optimal system offering both efficient bilayer formation and efficient ion transportation through the underlying film, either through the channels of MPTF or through the polymer network of the CTF. The motivation for using MPTF was that formation of a membrane protein containing SLB has been shown to be efficient on such substrates,^21^ whereas CTF could potentially utilize both the advantage of silica as well as the conducting characteristics of PEDOT:PSS.^18,19^ The quality of the SLB formation process was evaluated by using a quartz crystal microbalance with dissipation (QCM-D) monitoring, total internal reflection fluorescence (TIRF) microscopy, and fluorescence recovery after photobleaching (FRAP) for membranes made of pure synthetic lipids with or without the reconstituted membrane protein β-secretase 1 (BACE1) as well as cell-derived native lipid vesicles containing overexpressed BACE1.

## Experimental Section

### Materials

The lipids 1-palmitoyl-2-oleoyl-*sn*-glycero-3-phosphocholine (POPC), 1,2-dipalmitoyl-*sn*-glycero-3-phosphocholine (DPPC), 1-palmitoyl-2-oleoyl-*sn*-glycero-3-phosphoethanolamine (POPE), 1-palmitoyl-2-oleoyl-*sn*-glycero-3-phospho-(1′-*rac*-glycerol) (POPG), and *N*-(lissamine rhodamine B sulfonyl)-1,2 dioleoyl-*sn*-glycero-3-phosphoethanolamine (LRB-DOPE, λ_exc_/λ_em_ = 560/580 nm) were purchased from Avanti Polar Lipids Inc. (Alabaster, AL). Sodium chloride (NaCl), sodium dodecyl sulfate (SDS), dimethyl sulfoxide (DMSO), (3-glycidyloxypropyl)trimethoxysilane (GOPS), tetraethyl orthosilicate (TEOS, 98%), poly(ethylene glycol)_20_–poly(propylene glycol)_70_–poly(ethylene glycol)_20_ (P123), hydrochloric acid (HCl, 34%), acetone, 2-propanol (IPA), and chloroform (CHCl_3_) were purchased from Sigma-Aldrich, Germany. Phosphate buffered saline (PBS) was prepared from tablets (NaCl 137 mM, KCl 2.7 mM, phosphate buffer 10 mM, pH 7.4), also from Sigma-Aldrich. Poly(3,4-ethylenedioxythiophene)–poly(styrenesulfonate) (PEDOT:PSS) aqueous solution (Clevios PH1000) was purchased from Heraeus Precious Metals GmbH, Germany. Ethanol (99.7%) was purchased from Solveco. Borosilcate coverslips (ø 25 mm, No. 2) were purchased from VWR and QCM-D crystals from Q-Sense, Sweden. Bindzil 40/130 was a gift from Akzo Nobel, Sweden.

### Thin Film Fabrication

#### Conducting Polymer Thin Film

Borosilicate coverslips and SiO_2_-coated AT-cut quartz crystals for QCM-D were sonicated for 2 min in a 10 mM SDS solution, followed by ethanol and Milli-Q water (Millipore, France), then rinsed in Milli-Q, blow-dried with compressed nitrogen, and finally placed in a UV/ozone oven (ProCleaner Plus, BioForce Nanosciences) for 5 min. The PEDOT:PSS dispersion (Clevios PH1000) was filtered through a 0.2 μm filter (Millipore), mixed with GOPS (0.1% v/v) to promote adhesion to the substrate, and doped with DMSO (5% v/v). The polymer dispersion was deposited onto either the coverslips or the QCM-D sensors by spin-coating at 4000 rpm for 40 s (Spin150, SPS-Europe) followed by oven baking at 130 °C for 15 min.

#### Composite Thin Film

A PEDOT:PSS/silica nanoparticle composite thin film was spin-coated on top of the polymer film. The 25 nm silica nanoparticles (Bindzil 40/130) were filtered through a 0.1 μm filter (Whatman) and mixed with filtered PEDOT:PSS. Four dispersions were prepared with varied polymer/silica particle concentrations (Table 1). The dispersions were deposited onto substrates already coated with a conducting polymer thin film by spin-coating at 4000 rpm for 50 s followed by baking at 100 °C for 10 min.

**Table 1:** Composition of PEDOT:PSS/Silica Dispersions for Composite Thin Film Fabrication

sample	Bindzil 40/130 (% v/v)	Clevios PH1000 (% v/v)	water (% v/v)
CTF1	6.5	26.5	67
CTF2	10	23	67
CTF3	13	20	67
CTF4	16.5	16.5	67

#### Mesoporous Silica Thin Film

As an alternative substrate, mesoporous silica thin films were spin-coated on top of the polymer film, following a modified protocol of Alberius et al.^21,22^ In brief, 433 mg of TEOS was mixed with 500 mg of EtOH (99.7%) followed by addition of 225 mg of 0.01 M HCl and subsequent stirring at 300 rpm for 20 min. A 115 mg sample of P123 was dissolved in 500 mg of EtOH and then added dropwise to the TEOS solution followed by 20 min stirring at 300 rpm. The silica precursor solution was deposited onto either bare substrates or substrates with conducting polymer thin films by spin-coating at 4000 rpm for 60 s followed by aging for 24 h at 20 °C. The templating agent was removed through calcination by ramping 1 °C from 20 to 400 °C and continuing at 400 °C for 4 h. For the thin films deposited on top of a conducting polymer layer, alternative procedures for template removal were investigated to preserve the conductive polymer. The thin films were either (i) placed in a UV/ozone chamber for 30 min, (ii) immersed in ethanol with subsequent shaking for 24 h, either at room temperature or at 60 °C, followed by excess rinsing in ethanol and water, or (iii) immersed in methanol and sonicated for 5 min, followed by excess rinsing in methanol and water.

### Thin Film Characterization

The surface morphology was imaged with scanning electron microscopy (SEM) using a Leo Ultra 55 FEG SEM operated a 1–2 kV and a working distance of 1.7–2.2 mm. For thickness measurements, the samples were scratched, and the resulting step size was measured with an atomic force microscope (AFM) (Dimension 4100, Veeco, Plainview, NY). Step sizes were measured for 10 traces, evenly distributed between center and edge of the sample, by using the open source software Gwyddion. Root-mean-square roughness calculations were performed with Gwyddion for 2 μm by 2 μm AFM scans.

The electrical properties of PEDOT:PSS were investigated by using cyclic voltammetry (CV). The working electrode area was defined by a PDMS well placed on top of the measured film. PBS buffer was used as electrolyte, and a platinum mesh served as the counter electrode. Carbon paste (DuPont) was painted onto the films for enhanced electrical contact. Cyclic voltammograms were recorded with a Bio-Logic SP-200 potentiostat (Bio-Logic Science Instruments) at a scan rate of 50 mV/s. The potentials were set versus an Ag/AgCl reference electrode.

IR attenuated total reflectance (IR-ATR) spectra were obtained in a vacuum with an FTIR instrument (IFS-66v from Bruker) by using a single reflection ATR unit with a diamond crystal (Golden Gate from Graseby Specac). A clean glass slide was used as a reference to compensate for the absorption contributed by the substrate of the films.

### Vesicle Preparation

#### Preparation of Synthetic Lipid Vesicles

The vesicle compositions in the experiments were 100 mol % POPC, 100 mol % DPPC, 1 mol % LRB-DOPE with 99 mol % POPC (Rho-POPC), and 1 mol % LRB-DOPE with 99 mol % DPPC (Rho-DPPC). The lipids were dissolved in chloroform in a round-bottom flask and were exposed to a stream of N_2_ to evaporate the solvent and form a thin lipid film on the wall of the flask. Residual solvent was removed under vacuum, 90 kPa, for >1 h. The lipid film was rehydrated in PBS pH 7.4 by repeated vortexing to a final concentration of 1 mg/mL. The lipid solution was extruded 11 times through a 100 nm polycarbonate membrane (Whatman, UK) by using a mini extruder (Avanti Polar Lipids, USA). The lipid vesicle suspensions were stored at 4 °C. Prior to each experiment the lipid vesicles were diluted with PBS buffer to a total lipid concentration of 0.2 mg/mL.

#### Reconstitution of BACE in Proteoliposomes (BACE1pl)

POPC, POPE, and POPG were dissolved in chloroform, mixed in a 3:1:1 ratio, and dried under vacuum (>1 h). The lipid film was rehydrated in PBS pH 7.4 with 10 mM Triton-X100, and the mixed micelles were stored at 4 °C. Detergent-solubilized flBACE (0.05 mg/mL) was mixed with the micelles in a protein:lipid ratio of 1:3000 (a 150 nm liposome contains ∼200000 lipids) before addition of MQ-washed Biobeads SM2 (Bio-Rad) and incubated overnight with gentle mixing. The reconstitution process was monitored by dynamic light scattering (APS zetasizer, Malvern). The reconstitution efficiency was estimated by using the enzymatic assay assuming that nonreconstituted BACE1 was inactive. Successful incorporation of BACE1 was verified with fluorescence polarization (FP) measurements by using a Pherastar plate reader (BMG Labtech) with appropriate filter set (540/590 nm). The polarization of the rhodamine-labeled substrate analogue (30 nM) was measured in the presence of proteoliposomes (5 mg/mL) with or without addition of the unlabeled substrate analogue (2 μM) or in empty liposomes.

#### Preparation of Native Membrane Vesicle Hybrids (hNMV)

Native membrane vesicles (NMV) were prepared as described by Pace et al.^5^ In brief, cells (Sf9 or Sf21) were infected with Baculovirus containing full-length BACE1. A cell pellet was collected, and the cells were lysed and disrupted. Unbroken cells, mitochondria, nuclei, and soluble proteins were removed by centrifugation. The washed membrane pellet was dissolved in PBS and 20% glycerol. Aliquots of the NMVs were snap frozen in liquid N_2_ and stored at −80 °C until use. Native membrane vesicle hybrids (hNMV) were prepared by merging NMVs and POPC vesicles (1:2 mass ratio) by sonication.

### Characterization of Supported Lipid Bilayers (SLBs)

#### Preparation of PDMS Wells

Wells of polydimethylsiloxane (PDMS, Sylgard 184, Dow Corning, Midland, MI) were made by casting a sheet (3 mm thick), which was cut to fit the coverslips, and a hole punch was used to create wells in the PDMS slabs.

#### Total Internal Reflection Fluorescence Microscopy (TIRFM)

SLB formation was studied with TIRFM by using an inverted Nikon Eclipse Ti-E microscope (Nikon Corporation, Japan) equipped with an Andor Ixon+ camera (Andor Technology, Ireland), a 60× or 100× magnification oil immersion objective, a mercury lamp, a TRITC filter cube, and a perfect focus system. Micrographs (0.22 or 0.13 μm/pixel) were acquired in time-lapse mode with an exposure time of 100 ms. With TIRFM, the exponentially decaying evanescent field only excites fluorescently labeled vesicles in close proximity to the surface (∼100 nm); thus, fluorescent vesicles in the bulk are not detected. Fluorescence recovery after photobleaching (FRAP) was used to determine the diffusivity of LRB-DOPE in the lipid bilayer. A diode pumped solid-state 532 nm laser (B&W TEK Inc.) with 100 mW output was used to create a bleached area. The acquired images were analyzed by using the Hankel transform method, previously developed by Jönsson et al.^23^ A single exponential with an offset was fitted to the data yielding the diffusivity and mobile fraction. The vesicle compositions in these experiments were 100 mol % Rho-POPC, 33 mol % Rho-POPC with 67 mol % BACE1pl, and 33 mol % Rho-POPC with 67 mol % hNMV. A multiwelled PDMS slab with a well size of 3 mm in diameter and in height was attached on either a cleaned thin film surface or a bare coverslip, and a 10 μL vesicle solution was added into each well. The solutions were then incubated for 10 min and rinsed 10 times with 50 μL of PBS buffer.

To monitor the SLB formation, a small fraction (0.2% w/w) of rhodamine-labeled tracer vesicles (Rho-POPC) were mixed with unlabeled POPC vesicles, BACE1pl, or hNMV. The solution was composed of 1 μL of Rho-POPC vesicles (1 μg/mL), 2 μL of unlabeled vesicles (1 mg/mL), and 7 μL of PBS. The exact concentration of BACE1pl and hNMV stocks were unknown but estimated to be ∼1 mg/mL. The substrates with prepared thin films were rinsed in SDS and MQ and dried in N_2_, followed by UV/ozone treatment for 2 min. Bare borosilicate coverslips, used for control experiments, were boiled in 1% Liquinox (Alconox) in MQ for 30 min, thoroughly rinsed, and stored in water until use. A multiwelled PDMS slab was attached on either a cleaned thin film surface or a bare coverslip, and 10 μL vesicle solutions were added into the wells. The bilayer formation was recorded followed by rinsing with PBS to remove weakly bound vesicles.

#### Quartz Crystal Microbalance with Dissipation (QCM-D) Monitoring

Bilayer formation was also studied with QCM-D^8,9,24^ by using a Q-Sense E4 instrument (Biolin Scientific/Q-sense, Göteborg, Sweden). The technique is described elsewhere.^25^ SiO_2_-coated, composite thin film-coated, or mesoporous silica thin film-coated AT-cut quartz crystals were cleaned and immediately placed in the QCM-D flow chamber. The baseline was acquired in PBS buffer at 22 °C followed by addition of lipid solution (0.2 mg/mL) at a flow rate of 100 μL/min. After completed SLB formation or completed surface coverage, rinsing with PBS buffer was performed. The vesicle compositions in these experiments were 100% POPC, 100% DPPC, or various molar ratios of the two vesicle solutions.

## Results and Discussion

### Fabrication and Characterization of Thin Films

**Figure 1:**
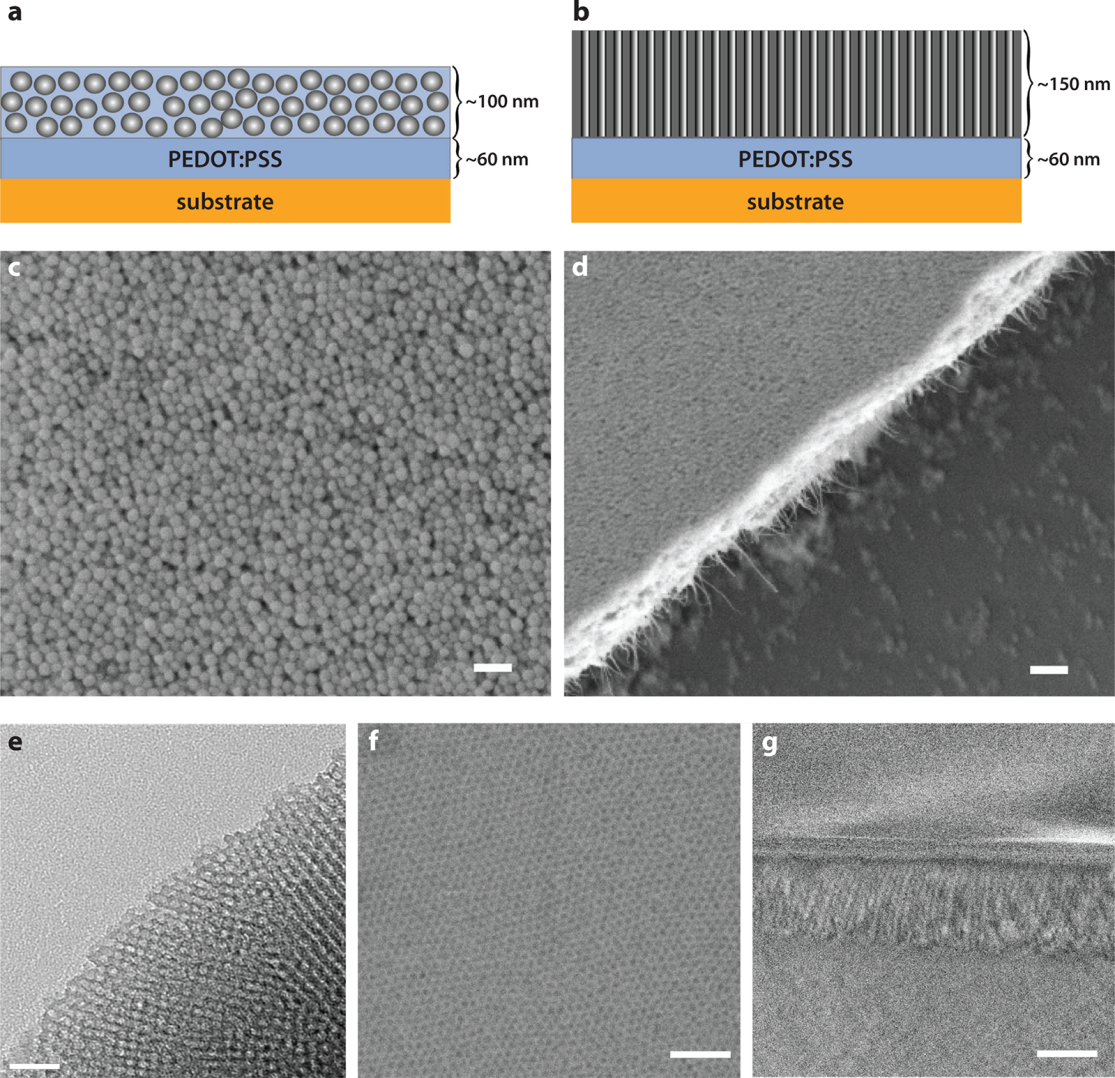
Two approaches to vesicle fusion promoting films on PEDOT:PSS. Schematic illustrations of the two platforms with (a) PEDOT:PSS/silica composite thin film (CTF) and (b) mesoporous thin film (MPTF) as top layer. SEM micrographs of CTF3 viewed (c) from top and (d) from the side, with the CTF in the left part of the image. Scale bars in (c) and (d) are 100 and 200 nm, respectively. (e) TEM micrograph of the MPTF showing a hexagonally ordered pore structure with a pore diameter of ∼7 nm. Scale bar: 50 nm. (f) SEM micrograph showing a top view of the MPTF with pores accessible from the surface. Scale bar: 100 nm. (g) SEM micrograph showing a cross section of a MPTF with a thickness of 150 nm deposited on a glass slide seen in the top of the image. Scale bar: 100 nm.

Two substrates were compared as potential platforms for supported lipid bilayers derived from vesicles of various compositional complexity, including pure phospholipid vesicles (liposomes), phospholipid vesicles with reconstituted membrane proteins (proteoliposomes), and vesicles derived from native cell membranes. The two platforms are illustrated in the schematics in Figure 1.

#### Composite Thin Films

In the first approach, composite thin films (CTF) of PEDOT:PSS and solid silica nanoparticles (∼25 nm) with varied particle/polymer composition (Table 1) were spin-coated on top of a pure PEDOT:PSS film (Figure 1a). The final concentrations of PEDOT:PSS, based on the solid content of polymer and silica in the dispersions, as well as thicknesses for the different thin films are presented in Table 2. The thickness of the underlying pure PEDOT:PSS film was consistent throughout the sample, while some of the composite thin films made from PEDOT:PSS and silica particles were thicker in the central regions of the films than in the outer. All CTFs showed very similar features in terms of topography and roughness, irrespective of particle polymer composition. The composite surfaces had an RMS roughness of 7 nm, with the difference between the highest and lowest points of the AFM micrograph being 60 nm. A representative AFM micrograph of the surface topography is shown in Figure S1. A representative scanning electron microscopy (SEM) micrograph of the composite film (CTF3) displays tightly packed silica particles (Figure 1c) with a network of polymer integrated in the film (Figure 1d). It was observed, however, that the film with lowest polymer concentration (CTF4) in the upper layer was more prone to cracking (Figure S2), suggesting that the polymer network is crucial for retaining the integrity of the film.

**Table 2:** Thicknesses of Silica Composite Thin Films (CTFs)

film type	PEDOT:PSS concn (%)	mean thickness (nm)	std dev (nm)
PEDOT:PSS	100	64	1
CTF1	9.0	86	8
CTF2	6.0	89	3
CTF3	4.0	113	18
CTF4	2.4	145	5

#### Mesoporous Silica Thin Film

In the second approach, a mesoporous silica thin film (MPTF) was spin-coated on top of a pure PEDOT:PSS film (Figure 1b). The TEM and SEM micrographs of the mesoporous silica film display hexagonally arranged pores accessible from the surface, with a uniform pore size around 7 nm (Figure 1e,f). A cross-section view of the film displays an ∼150 nm film thickness with pores standing in the upright position (Figure 1g).

**Figure 2:**
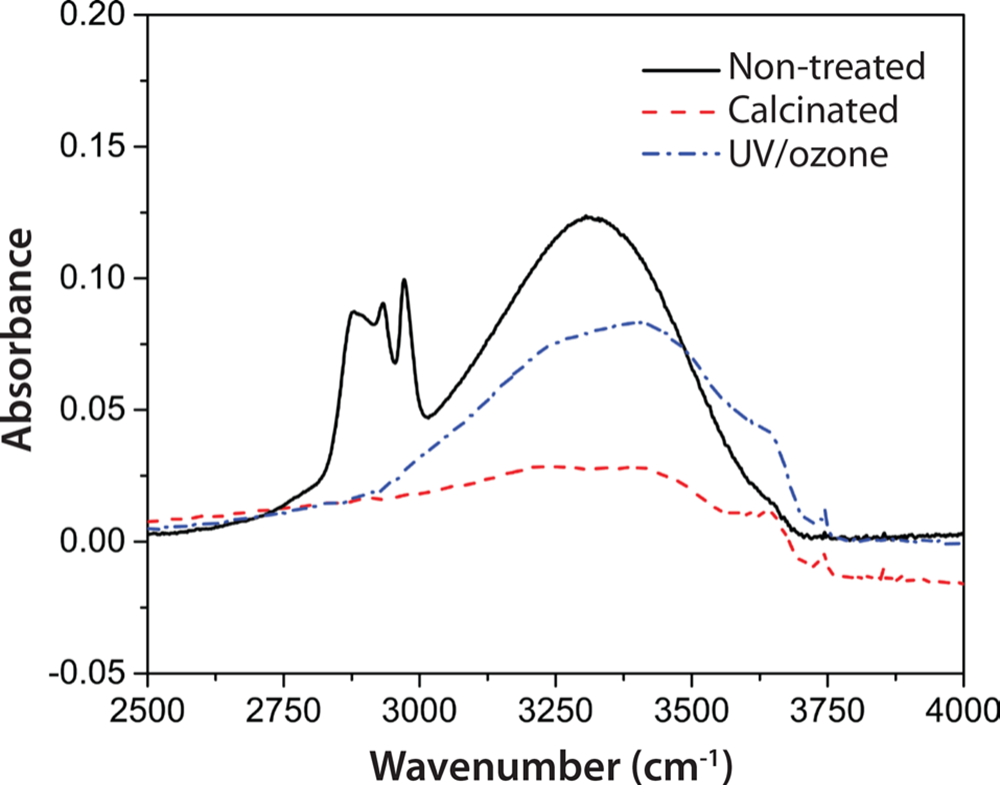
IR-ATR absorbance spectra of nontreated, calcinated, and UV/ozone-treated mesoporous thin films (MPTFs) on glass.

To remove the structure-directing agent from the interior of the pores without damaging the conducting polymer various strategies were tested, including UV/ozone treatment and extraction in either ethanol or methanol. IR-ATR spectra of calcinated mesoporous thin films without surfactant and untreated films with surfactant present in the pores were compared with the treated films (Figure 2). For these measurements, mesoporous thin films were spin-coated directly onto glass substrates without the pure PEDOT:PSS layer in between (to avoid complicating the spectra with additional organic molecules). The spectral bands around 2800–3000 cm^–1^ represent the CH stretching in organic compounds serving as an indicator of the presence/absence of surfactant in the films since these features are associated with the surfactant alone. As can be seen in the graph, the untreated sample has a well-defined peak in this region whereas the calcinated sample, without surfactant, completely lacks this peak. The UV/ozone-treated film also lacks this peak, which strongly indicates that the surfactant hydrocarbons have been removed. The bands between 3000 and 4000 cm^–1^ are characteristic of OH stretching pertaining to hydroxyl groups from both the surfactant polymer chains and the silica pore surface. The total area of this band region is lower for the UV/ozone-treated sample compared to the untreated sample, being attributed to removal of the hydroxyl groups that belong to the surfactant. The OH stretching from the untreated sample has a Gaussian-like profile of the bond lengths, whereas the UV/ozone treated sample (i.e., surfactant removed) comprises different subsets of hydroxyl groups, as evidenced by the shifted profile. In particular, some are essentially not involved in hydrogen bonding any longer (3750 cm^–1^), probably those located on the surface of the pores. Other hydrogen bonds are less strong as they are shifted to higher wavenumbers compared to the untreated sample. Calcination not only affects the surfactant but also reduces the area of the OH stretching attributed to the hydroxyl groups on the silica pore walls as they transform into bridging siloxane groups. The OH profiles of the untreated and UV/ozone-treated films are therefore not completely comparable with the calcinated film. The IR-ATR spectra for the ethanol- and methanol-treated films were almost identical to the untreated films which indicate that these treatments did not extract the surfactant successfully (spectra not shown).

#### Ion Transport

**Figure 3:**
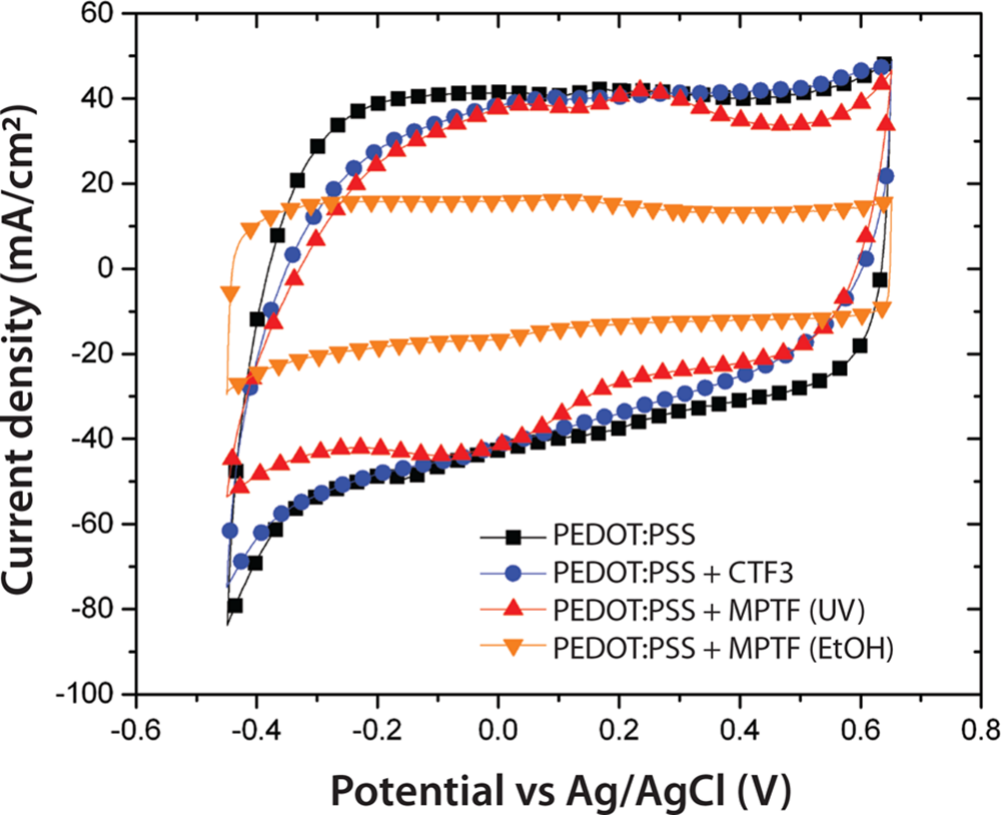
Cyclic voltammograms of bare PEDOT:PSS and PEDOT:PSS with three different top layers. The layers are a composite thin film (CTF3), UV/ozone-treated mesoporous silica thin film (MPTF), and mesoporous silica where the surfactant was extracted with ethanol at room temperature.

Maintained ion exchange between the PEDOT:PSS film and the electrolyte is essential to eventual incorporation of the proposed material systems in biosensors and other bioelectronic applications. Cyclic voltammetry (CV) was performed to investigate the ion transport through the thin films, and the resulting voltammograms are presented in Figure 3. CTF3 is shown here due to the fact that, first, the underlying PEDOT:PSS should dominate the CV compared to the smaller contribution from material in the CTF pores and, second, that CTF3 was indicated as optimal for SLB formation based FRAP and QCM experiments below. The voltammograms recorded for the CTF3 and MPTF with UV/ozone treatment display current density levels similar to the levels obtained for bare PEDOT:PSS, indicating that ions can effectively penetrate into the bulk of the film and contribute to the large double-layer capacitance typically observed for PEDOT:PSS. Upon scan direction reversal (at the positive and negative extremes of the voltage sweep), the current slopes were less steep when a composite top layer was added, which is an indication that the resistance in the system has increased. This is likely due to the top layer limiting the ion transport to the polymer. Other CTF compositions showed similar voltammograms to the one shown in Figure 3. For the MPTF, CV was also performed to determine whether the different surfactant removal methods affected the electrical properties of the PEDOT:PSS bottom layer. When the surfactant was removed through UV/ozone treatment, current densities were on similar levels as bare PEDOT:PSS. Upon scan direction reversal, the current slopes were less steep, which—in analogy with the CTF systems—is likely due to the top layer limiting the ion transport to the polymer rather than damage done to the polymer itself. In the case of MPTF with ethanol extraction, the current density was reduced, and the current changed rapidly when the scan direction was reversed. This capacitive behavior could be explained by incomplete extraction of surfactants leaving the pores partially or completely closed. In this case, the more ideal capacitive behavior (i.e., more rectangular CV), in conjunction with the lower current density, indicates capacitive charging of the outer layer of the MPTF itself rather than the underlying PEDOT:PSS. Extraction with ethanol at elevated temperature and methanol generated voltammograms of the same shapes as the room temperature ethanol extraction shown here. In regard to the integrity of the underlying conducting polymer, PEDOT:PSS was used as the working electrode without a metal bottom electrode. Because the current density levels were largely unaffected by the additions of CTF3 and MPTF with UV/ozone treatment, we conclude that the underlying PEDOT:PSS remains sufficiently conductive to act as an electrode.

### Supported Lipid Bilayer Formation

As a first evaluation of the two substrates as potential supports for bilayer structures, pure lipid vesicles were deposited onto the surfaces, and bilayer formation was characterized by using TIRF microscopy, FRAP, and QCM-D.

#### SLB Mobility Measurements

**Figure 4:**
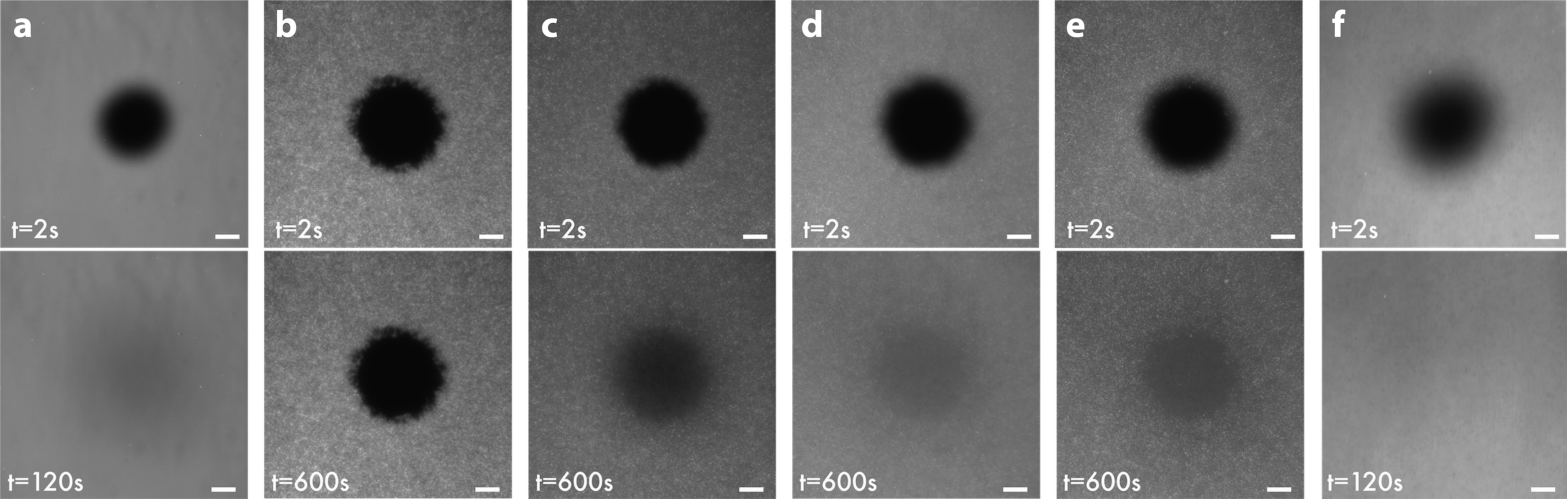
FRAP micrographs showing the recovery of Rho-POPC SLBs on (a) glass, (b) CTF1, (c) CTF2, (d) CTF3, (e) CTF4, and (f) MPTF. Scale bar: 40 μm. Data correspond to Table 3.

The formation and quality of supported lipid bilayers were investigated by using fluorescence recovery after photobleaching (FRAP).^26,27^ FRAP provides information about the lateral diffusion of fluorescently labeled lipids as the recovery in fluorescence intensity of a defined irreversibly photobleached region is monitored. Vesicles were deposited on a bare glass substrate (control), composite thin films with varying PEDOT:PSS/silica ratios, and mesoporous silica thin films. The modeled lipid diffusivity (*D*) and recovery/mobile fraction (*R*) are shown in Table 3. The SLB formed when depositing Rho-POPC vesicles on the glass substrate showed lipids with a lateral diffusivity (*D* = 1.55 μm^2^/s) and full recovery (*R* = 99.7%) (Table 3). In Figure 4a, the top image shows a snapshot of the SLB recovery 2 s post-photobleaching whereas the image just below shows the recovery after 120 s. The two images display a smooth surface with very few unruptured vesicles.

**Table 3:** Fluorescence Recovery after Photobleaching Data of Rhodamine Labeled POPC Lipids after Vesicle Deposition on Various Substrates (Data Correspond to Figure 4)

	Rho-POPC	
substrate	diffusivity (μm^2^/s)	recovery (%)
glass	1.55 ± 0.01	99.7 ± 0.2
CTF1		
CTF2	0.17 ± 0.05	84.5 ± 3.0
CTF3	0.47 ± 0.06	95.4 ± 2.5
CTF4	0.53 ± 0.04	86.2 ± 1.6
MPTF	2.07 ± 0.02	99.3 ± 0.3

A dramatic decrease in *D* and *R* was observed for bilayers on the composite thin films compared to the glass substrate. For CTF1, with the highest PEDOT:PSS content, a mottled surface was observed, indicating a large number of unruptured vesicles. A comparison between the photobleached area at 2 and 600 s (Figure 4b) showed no difference in recovery, further indicating an unsuccessful bilayer formation where the vesicles adsorb to the surface without rupturing. As the PEDOT:PSS content was decreased, the lipid diffusivity increased. A rather slow but detectable diffusivity of 0.17 μm^2^/s and a fairly low recovery of 84.5% were observed for the CTF2 substrate (Figure 4c). A high abundance of unruptured vesicles on the surface was also detected. A significant improvement was observed for CTF3 and CTF4 (Figure 4d,e) with a slightly higher diffusivity (but lower recovery) on CTF4 (CTF3: *D* = 0.47 μm^2^/s and *R* = 95.4%; CTF4: *D* = 0.53 μm^2^/s and *R* = 86.2%). As discussed above in the Fabrication section, the low polymer content in CTF4 caused small cracks in the film which likely inhibited full recovery of the SLB. The decreasing bilayer diffusivity with increasing polymer content is attributed to the hydrophobic nature of PEDOT, known to prevent vesicle rupture and lipid diffusion,^18^ as well as its positive charge, which is likely to increase electrostatic attraction to the lipid head groups. In addition to the PEDOT hydrophobicity, the overall slower diffusion of fluorescently labeled lipids on composite thin films could partly be explained by a larger surface roughness of the spin-coated thin films compared to a glass substrate (see sections on Fabrication and Characterization). However, an RMS roughness of 7 nm, with the difference between the highest and lowest points of the image being 60 nm (Figure S1), should only have a minor impact on the lipid diffusion. Moreover, the low concentration of PEDOT:PSS together with heat treatment of the thin film during manufacturing prevents the film from swelling when exposed to buffer and would therefore not significantly influence an increased surface roughness (film swelling is addressed in more detail below). CTF3 was chosen over CTF4 for the subsequent studies with more complex vesicle structures due to the higher concentration of PEDOT:PSS together with better SLB recovery.

When forming SLBs on mesoporous silica thin films (MPTFs), the resulting diffusivity was higher compared to the composite thin films and even to that observed for SLBs on glass (*D* = 2.07 μm^2^/s, *R* = 99.3%, Figure 4f). This has been observed previously by Isaksson et al. in studies of SLBs containing human aquaporin proteins on mesoporous silica thin films,^21^ and is attributed to reduced interaction between substrate and lipid by the porous architecture. The FRAP micrographs show a smooth SLB surface with very few unruptured vesicles, and the photobleached area is completely recovered after 120 s.

#### Monitoring Bilayer Formation with QCM-D

**Figure 5:**
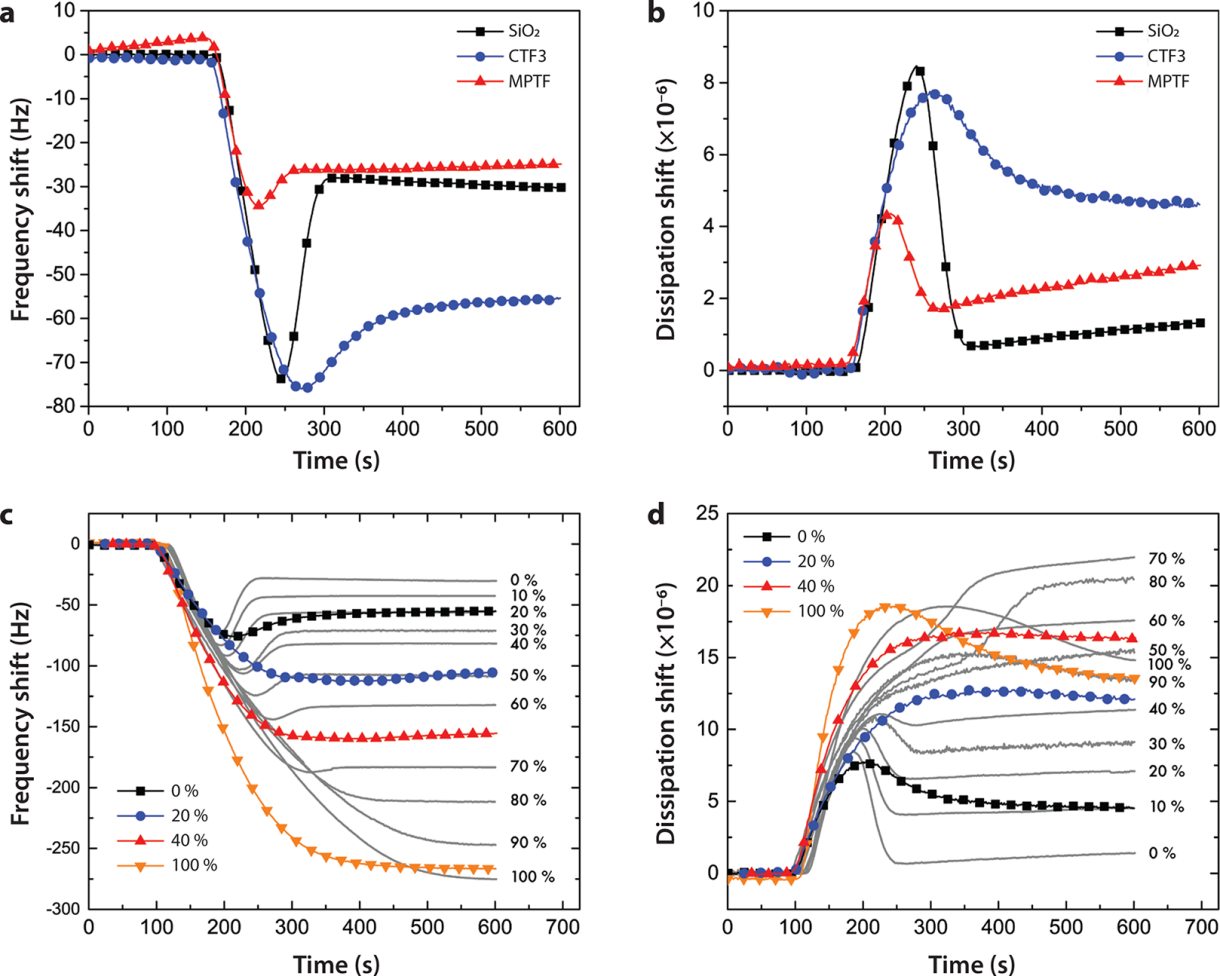
QCM-D investigation of vesicle adsorption. (a) Frequency and (b) and dissipation (fifth overtone) shifts showing the deposition of POPC vesicles on a SiO_2_-, CTF3-, or MPTF-coated sensor. (c) Frequency and (d) dissipation showing adsorption of various ratios of POPC/DPPC on CTF3-coated (colored) and a SiO_2_-coated (gray) sensor. Percentages in legend indicate percent DPPC.

CTF3 composite thin films were fabricated directly on top of a QCM-D sensor and compared to typical bilayer formation on a SiO_2_-coated sensor.^8,9,24,28^ Prior to bilayer formation, thin film swelling was investigated by flowing PBS through the chamber for 60 min. Because a stable baseline was observed and no frequency or dissipation drift was detected, it was concluded that no liquid was taken up by the film. However, a significant baseline drift was observed if the heat treatment step was omitted during the preparation, suggesting that this step is necessary to form a robust composite thin film (results not shown). In fact, a slow baseline drift (both frequency and dissipation) could be observed after several hours, which is consistent with uptake of liquid and reduction in film rigidity. To monitor vesicle adsorption, a POPC vesicle solution (0.2 mg/mL) was fed through the chamber at a flow rate of 100 μL/min (Figure 5a,b). As POPC vesicles adsorbed to the surface, the frequency shift (Δ*f*) decreased due to mass increase while the dissipation shift (Δ*D*) increased until a critical concentration of adsorbed vesicles was reached. At the critical vesicle coverage, they started to rupture into a bilayer, resulting in release of coupled liquid accompanied by an increase in Δ*f* and a decrease in Δ*D*, signaling formation of a more rigid bilayer. Vesicle adsorption and rupture are not completely separate processes but take place simultaneously once a critical surface concentration (CSC) is obtained.

Typical frequency and dissipation profiles for a planar high quality lipid bilayer on a SiO_2_-coated sensor are shown in Figure 5a,b, with final Δ*f* and Δ*D* values (measured at the fifth overtone) of −28 Hz and 0.6 × 10^–6^, respectively, which is in agreement with previous results.^24,28^ The general POPC vesicle deposition profile on a CTF3-coated sensor was quite similar to a SiO_2_-coated sensor in terms of Δ*f* minima and Δ*D* maxima (Figure 5a,b and Figure S3a), and the increase in Δ*f* and decrease in Δ*D* after reaching a plateau at 270 s suggest vesicle rupture. However, the final Δ*f* and Δ*D* values of −55 Hz and 4.5 × 10^–6^, respectively, indicate an incomplete bilayer with coadsorbed unruptured vesicles. To determine the fraction of unruptured vesicles remaining on the surface, the QCM-D profile for the deposition of POPC vesicles was compared with the profile of DPPC vesicles as well as solutions containing various ratios of vesicles made of either POPC (which are prone to rupture) or DPPC (which do not rupture) (Figure 5c,d). DPPC lipids have a higher melting temperature than POPC lipids and do not normally form bilayers at ambient temperature. The Δ*f* response upon adsorption of DPPC vesicles and mixtures of DPPC and POPC vesicles are shown in Figure 5c and Figure S3b. For DPPC vesicles, adsorption resulted in a continuous decrease in frequency until surface saturation was obtained at Δ*f* around −270 Hz (fifth overtone) for both a SiO_2_-coated and a CTF3-coated sensor. After saturation the frequency remained constant with no apparent vesicle rupture. The corresponding Δ*D* response also increased monotonically, which is consistent with formation of a viscoelastic layer of vesicles with liquid trapped within and between the vesicles (Figure 5d). After reaching a maximum, the dissipation slowly decreased as the vesicles were packed more tightly together and finally leveled out at 16 × 10^–6^ for a SiO_2_-coated sensor and 14 × 10^–6^ for a CTF3-coated sensor. The similar profiles for both frequency and dissipation demonstrate that a comparable number of vesicles were deposited onto the two surfaces, further indicating a similar available surface area for the adsorbed vesicles.

To verify that the profile of the POPC deposition onto a CTF3 substrate corresponded to a partially formed bilayer and to determine the fraction of unruptured vesicles, a series of experiments were performed with various ratios of POPC and DPPC vesicles deposited onto the substrates. The SiO_2_-coated sensor was used as a reference, with the percentage of DPPC vesicles in the mixture of POPC and DPPC vesicles representing the fraction of unruptured vesicles on the surface. As displayed in Figure 5c, a pure POPC solution (0% DPPC) deposited on the composite film corresponded well with 20% unruptured DPPC vesicles on a SiO_2_ surface. The dissipation was somewhat lower with a final Δ*D* at 4.5 × 10^–6^, which corresponded to 10% unruptured vesicles (Figure 5d), indicative of larger deformation of unruptured POPC on CTF3 than for DPPC on SiO_2_. When depositing a vesicle solution with 20% DPPC on the CTF3 film, the final Δ*f* corresponds to 50% unruptured vesicles whereas Δ*D* corresponds to 40% unruptured vesicles. This is indeed in fairly good agreement with the expected quantity of 40% unruptured vesicles. A similar behavior was observed when depositing a vesicle solution of 40% DPPC on CTF3 where the final Δ*f* and Δ*D* end up close to 60% unruptured vesicles on a SiO_2_ surface. As discussed above, 100% unruptured vesicles display similar frequency and dissipation profiles for both surfaces, which shows that in this case vesicles adsorb without rupturing on both surfaces.

The SLB formation profiles for POPC vesicles deposited on a MPTF-coated sensor indicate a complete bilayer with no unruptured vesicles on the surface with a final Δ*f* at −26 Hz and Δ*D* at 1.7 × 10^–6^ (Figure 5a,b), confirming the FRAP data above. The onset of SLB formation at a lower CSC for MPTF than SiO_2_ suggests that onset of vesicle rupture requires a lower degree of vesicle interactions on MPTF compared with SiO_2_. Considering the fact that the projected area of SiO_2_ is lower on MPTF than on planar SiO_2_, this is surprising, and suggests that local vesicle deformation induced near the sub-10 nm pores destabilizes the vesicle, which in turn promotes rupture and subsequent SLB formation.

#### Monitoring SLB Formation with Tracer Vesicles

**Figure 6:**
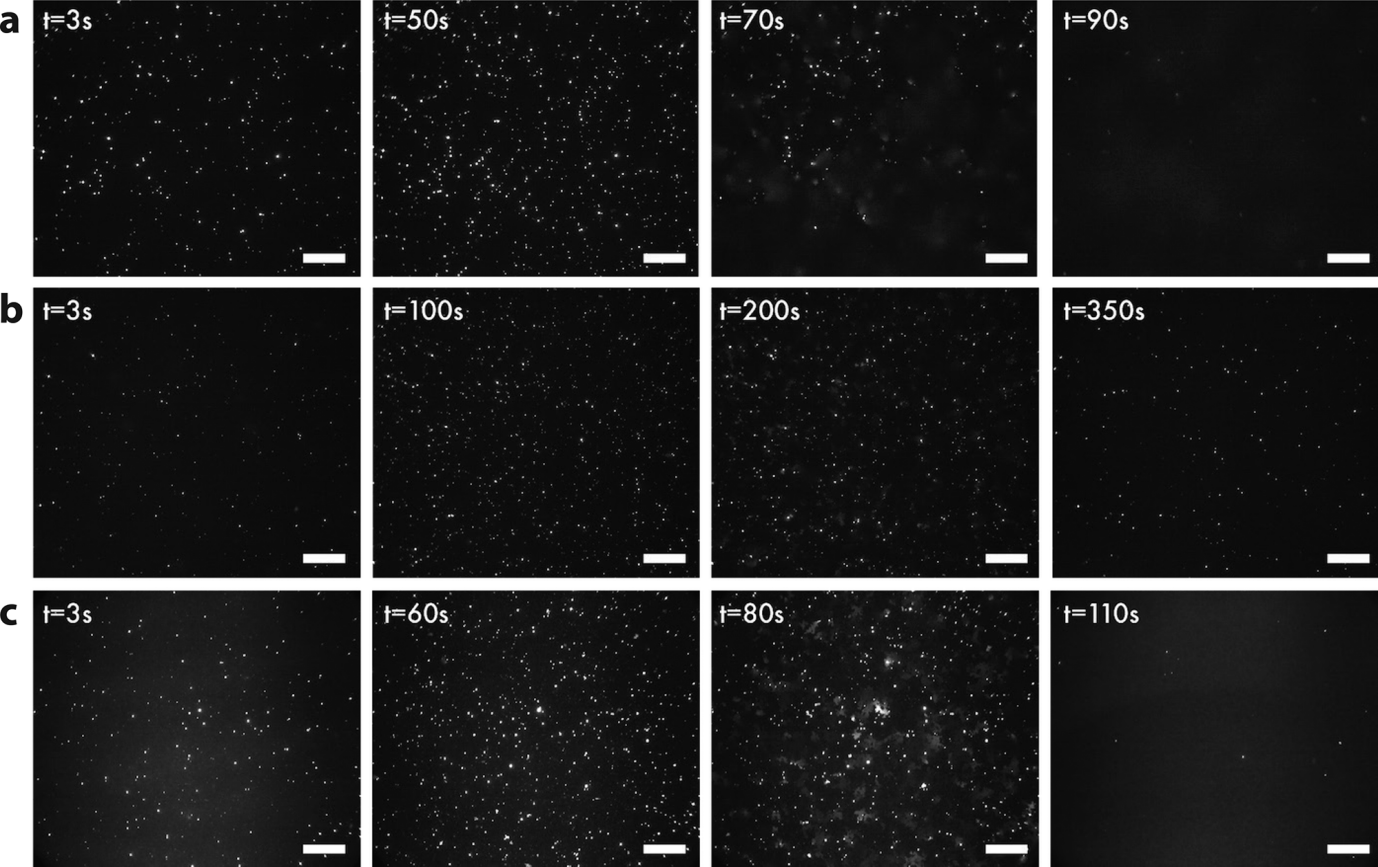
TIRFM micrographs showing SLB formation via adsorption and rupture of rhodamine-labeled POPC vesicles (tracers) together with unlabeled POPC vesicles on (a) glass, (b) composite thin film (CTF3), and (c) mesoporous silica thin film (MPTF) substrates. Scale bars: 40 μm.

**Figure 7:**
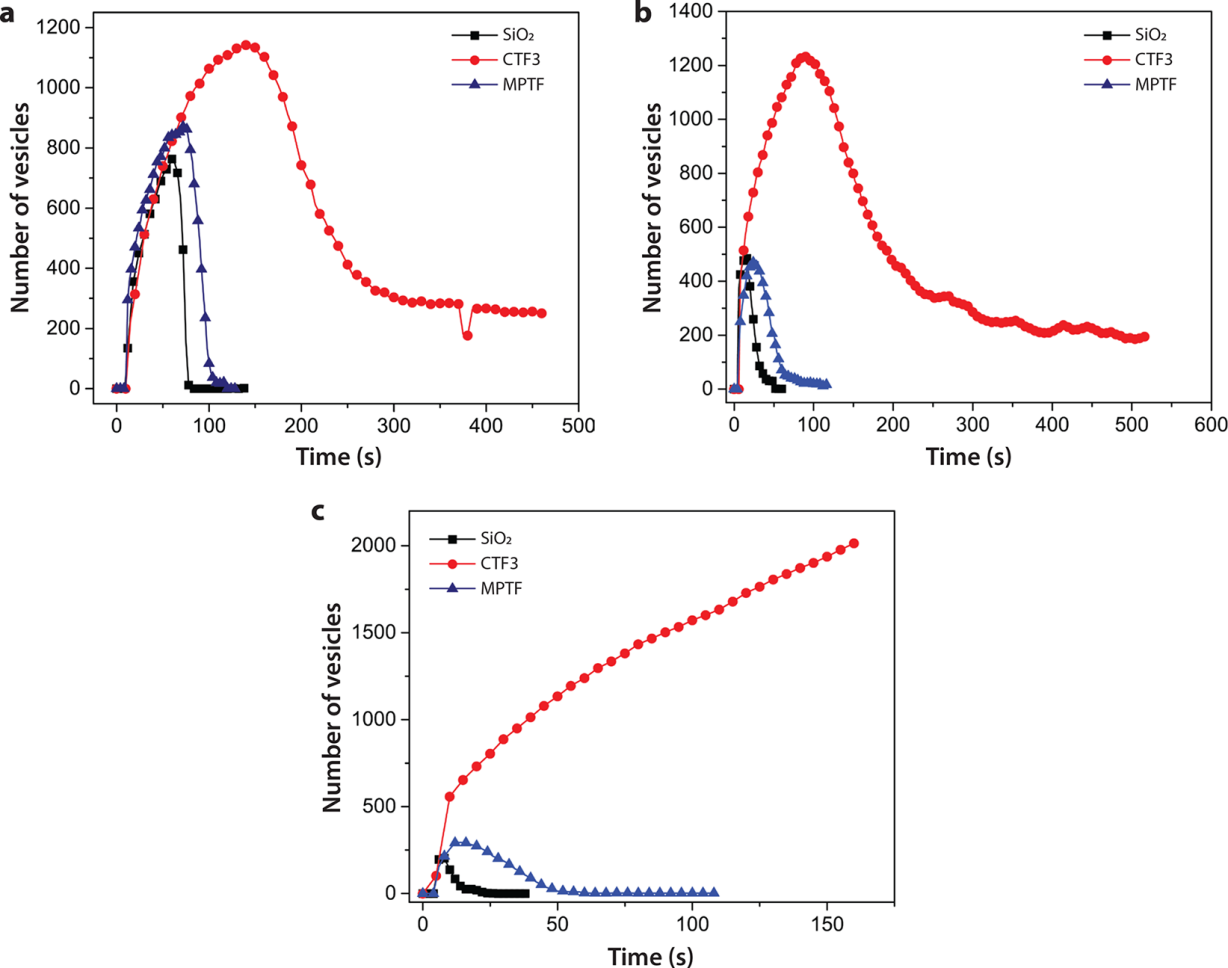
Number of adsorbed tracer vesicles as a function of time during bilayer formation within a 280 × 230 μm^2^ field of view on various substrates. Tracer vesicles mixed with (a) POPC, (b) BACE1pl, or (c) hNMV.

SLB formation was also monitored in real time with TIRF microscopy upon addition of unlabeled vesicles together with a small fraction (0.2% w/w) of rhodamine-labeled tracer vesicles (Rho-POPC) to the various substrates. Snapshots of the vesicle adsorption and subsequent SLB formation are shown in Figure 6, with only the small fraction of fluorescently labeled lipids being detected by TIRFM, while the majority of the vesicles (unlabeled) are present but invisible. The temporal evolution of number of unruptured tracer vesicles on the substrate is shown in Figure 7a. On a (SiO_2_-based) glass substrate, following adsorption the number of tracer vesicles on the surface increased until a CSC was reached at 60 s (Figures 6a and 7a), after which vesicle rupture was initiated and SLB formation occurred in a wave-like manner, with the number of vesicles and fluorescence intensity decreasing as the labeled lipids diffused from the ruptured vesicles into the bilayer. A continuous bilayer was fully formed after 90 s, displaying a surface nearly absent of unruptured vesicles.

The substrate-induced bilayer formation of pure POPC vesicles was significantly slower on a composite thin film (CTF3), and the CSC was higher compared to a glass substrate (Figures 6b and 7a). The number of vesicles on the surface continued to increase for about 150 s, and the subsequent bilayer formation propagated for another 150 s. The fraction of unruptured vesicles remaining on the surface after SLB formation was, according to Figure 7a, about 25%, which was unchanged after rinsing with buffer. This is comparable to the QCM-D results where the amount of unruptured vesicles were quantified to be ∼20% (see above). The surface topography of the composite film could affect the continuity of the bilayer, but it is more likely that patches of conductive polymer on the thin film surface prevent the propagation of a uniform fluid bilayer.

The POPC bilayer formation on a mesoporous silica thin film was comparable to the glass substrate but in this case with larger CSC (Figures 6c and 7a), which could indicate that the fluorescently labeled tracer vesicles do not behave in exactly the same manner as unlabeled vesicles. A continuous bilayer was fully formed after 100 s, displaying a surface nearly absent of unruptured vesicles after rinsing.

### Bilayers Derived from Complex Vesicle Structures

To provide a closer mimic of a natural biomembrane, vesicles with complex compositions were deposited onto the composite thin film (CTF3) and the mesoporous silica thin film (MPTF). A bare glass substrate was used as a reference. The vesicles were either proteoliposomes, containing POPC lipids with incorporated BACE1 transmembrane proteins (BACE1pl)^29^ or native membrane vesicle hybrids (hNMVs) derived from native membranes and POPC vesicles.^5^ Such vesicles have previously been shown to effectively transfer BACE1 to the resulting bilayer.^5,30^ The results of these studies are summarized in Table 4.

**Table 4:** Fluorescence Recovery after Photobleaching Data of LRB-DOPE Lipids after Deposition Together with BACE1Pl and HNMV on Glass, CTF3, and MPTF Substrates^a^

	Rho-POPC		BACE1pl + Rho-POPC (2:1)		hNMV + Rho-POPC (2:1)	
substrate	diffusivity (μm^2^/s)	recovery (%)	diffusivity (μm^2^/s)	recovery (%)	diffusivity (μm^2^/s)	recovery (%)
glass	1.55 ± 0.01	99.7 ± 0.2	1.48 ± 0.04	99.2 ± 0.1	0.98 ± 0.03	98.8 ± 0.3
CTF3	0.47 ± 0.06	95.4 ± 2.5	0.21 ± 0.11	97.4 ± 4.7		
MPTF	2.07 ± 0.02	99.3 ± 0.3	1.98 ± 0.05	99.4 ± 0.3	1.47 ± 0.01	99.5 ± 0.5

a The Rho-POPC results are duplicated from Table 3 for comparison.

#### Complex Bilayer Mobility

When depositing a mixture of proteoliposomes (BACE1pl) and Rho-POPC vesicles (2:1 mass ratio) on a bare glass substrate, both diffusivity and recovery (via TIRFM) were comparable to pure Rho-POPC (*D* = 1.48 μm^2^/s, *R* = 99.2%), indicating successful SLB formation with only a minor influence of the BACE1 transmembrane proteins on the bilayer formation and lipid diffusion. When instead mixing hNMVs with Rho-POPC vesicles (2:1 mass ratio, 22% NMV), a 34% reduction in lipid diffusivity along with an only slight drop in recovery (*R* = 98.8%) was observed in comparison to SLBs formed from pure Rho-POPC vesicles. These results were expected since gel-phase lipids, sterols, and membrane proteins in the hNMVs should lower the mobility of the fluorescent lipid probe (LRB-DOPE).^31^ Pace et al. previously reported a 70% reduction in lipid diffusion and a mobile fraction of 86% when forming SLBs from hNMVs containing Rho-POPC (1:2 mass ratio, 33% NMV) on a bare glass substrate.^5^ As our hNMV plus Rho-POPC SLB is only 22% NMV by mass instead of 33% NMV, it is likely that this difference in the amount of native membrane content is responsible for the observed differences in lipid diffusion. In contrast, the differences in mobile fraction most likely arise from the LRB-DOPE being integrated into the hNMVs in the work by Pace et al.,^5^ while in this work the hNMVs are coadsorbed with Rho-POPC vesicles. This means that most of the LRB-DOPE will in our case be released into the continuous SLB, rather than being compartmentalized in nonruptured hNMVs.

In contrast to the glass substrate, depositing a 2:1 BACE1pl/Rho-POPC mixture onto CTF3 resulted in a bilayer with a lower recovery and a drastically slower diffusion of fluorescently labeled lipids. This indicates that there is an unfavorable interaction between the SLBs components and the underlying CTF3 substrate. While the CTF3 substrate demonstrated a reduced lipid diffusion in comparison to bare glass for the lipid only Rho-POPC SLBs, diffusion was even further reduced upon the addition of BACE1pl. Because BACE1pl has a lower propensity to rupture due to their relatively high transmembrane protein content, the CTF3 surfaces which already perform worse than bare glass at forming even pure lipid SLBs may not be able to rupture the BACE1pls efficiently, thus rendering only the Rho-POPC vesicles to form the SLB.^32^ Alternatively, the reduction in diffusivity and recovery could also in part be due to stronger nonspecific interactions between the BACE1 transmembrane proteins and the composite substrate.^33^ These immobile proteins could in turn further obstruct lipid diffusion. A fluid bilayer could not be detected for the hNMV/Rho-POPC mixture on CTF3, further indicating that these surfaces do not react favorably enough with vesicles of complex composition to promote the production of high quality SLBs with good diffusive properties.

In contrast to the CTF3 substrate, lipid diffusion and recovery were more or less unchanged when depositing a 2:1 BACE1pl/Rho-POPC vesicle mixture onto a MPTF substrate compared to pure Rho-POPC vesicles (*D* = 1.98 μm^2^/s, *R* = 99.4% vs *D* = 2.07 μm^2^/s, *R* = 99.3%), again indicating successful SLB formation and minor BACE1 influence. The deposition of a 2:1 hNMV/Rho-POPC mixture resulted in a 26% reduction in lipid diffusivity but relatively unchanged recovery (*R* = 99.5%). The reduction in diffusivity was lower compared to the same type of SLB on a glass substrate (34%), indicating that there may be less attractive interactions between the SLB and the MPTF compared to bare glass. In addition, a mesoporous silica substrate might in fact be better for promoting SLB formation and enhanced mobility of its components even from native membrane vesicles.

Attempts to detect the mobility of the BACE1 protein after forming the bilayer were made by introducing a rhodamine-labeled BACE1 inhibitor peptide that binds to the active site of the ectoplasmic domain. Unfortunately, mobility measurements were not possible because of nonspecific binding of the peptide to PEDOT:PSS and porous silica.

#### Monitoring Complex SLB Formation with Tracer Vesicles

The time evolution pattern of the lipid bilayer formation with incorporated transmembrane proteins was monitored on glass, CTF3, and MPTF by mixing BACE1pl with Rho-POPC tracers (0.2% w/w). For both the glass substrate and MPTF, a rapid bilayer formation was observed with only a minor amount of unruptured vesicles remaining on the surface (Figure 7b). The critical surface concentration (CSC) was significantly larger for vesicles deposited on CTF3 with 15% unruptured tracer vesicles coadsorbed on the substrate. The relative differences in bilayer formation on the different substrates are in accordance with the SLB formation when depositing pure POPC vesicles (Figures 6 and 7a). Surprisingly, the CSC was lower on all substrates for tracer vesicles deposited together with BACE1pl compared to pure POPC vesicles. Traces of residual detergent may be retained in the sample after incorporation of the transmembrane protein. Detergent is known to destabilize lipid membranes and thereby lower the energy barrier for vesicle rupture, which could therefore also contribute to a more rapid SLB formation.^34^ Another possible explanation for the rapid SLB formation is a higher starting concentration of BACE1pl compared to POPC vesicles, as the exact concentration of the BACE1pl is not known after the detergent depletion steps during the proteoliposome preparation. Because a higher-than-estimated concentration of BACE1pl would result in a reduction of the fraction of tracer vesicles on the surface, this is one possible explanation to the somewhat lower amount of unruptured vesicles (15%) compared to a bilayer formed from pure POPC vesicles. It is also worth pointing out that during BACE1pl preparation a similar vesicle size was indeed obtained, but with a slightly broader size distribution compared to pure POPC vesicles. Even if larger vesicles must not necessarily deform and rupture more readily, they may occupy a larger surface area, which in would in turn contribute to a decreased CSC.^24^ The use of pure BACE1pl together with tracers, however, proves that a bilayer can be formed with primarily proteoliposomes and thus provides strong evidence that proteoliposome constituents are present in the bilayer formed from the 2:1 BACE1pl/Rho-POPC vesicle mixture use for the FRAP studies.

When depositing tracers mixed with hNMVs, successful bilayers with a minor amount of unruptured vesicles were confirmed on glass and MPTF substrates (Figure 7c). However, the bilayer formation was slower on MPTF and the CSC larger compared to the glass substrate. This was also observed for POPC vesicles and BACE1pl, although the difference was much smaller. Therefore, fast bilayer formation does not necessarily coincide with a faster lipid diffusion once the bilayer is formed, since the FRAP data show that the lipid diffusivity is higher on MPTF than on glass (Table 4). A possible explanation is that there is a stronger vesicle–surface interaction on the solid glass substrate compared to the porous silica surface. A stronger interaction would cause a larger vesicle deformation and thereby more strain, lowering the activation barrier for rupture and fusion^24^—but once the bilayer was formed, the lipids would diffuse more freely on top of a porous substrate. As the FRAP data show (Table 4), no bilayer formation was detected on CTF3, which can be seen in Figure 7c as a continuous increase in vesicle absorption. Surface saturation was reached after ∼600 s.

## Conclusions

In this work, we have investigated the vesicle fusion capabilities of PEDOT:PSS/silica composite films and mesoporous silica films coated on top of underlying PEDOT:PSS electrodes. We found that both types of films had the capacity to transport ions from the bulk (above the SLB) down to the PEDOT:PSS bottom layer, with minor reduced effect on the electrical properties of the pure polymer. In this way, the mixed ionic/electronic conducting capabilities of the PEDOT:PSS electrode could be preserved while preserving vesicle fusion with the added silica-containing layer.

Vesicle compositions of increasing complexity, ranging from pure phospholipid (POPC) vesicles to proteoliposomes with incorporated BACE1 transmembrane proteins (BACE1pl) and vesicles derived from native membranes (hNMV), were compared. The integrity of bilayers, formed through vesicle fusion, was investigated by using quartz crystal microbalance with dissipation (QCM-D) monitoring, total internal reflection fluorescence microscopy (TIRFM), and fluorescence recovery after photobleaching (FRAP). The lipid diffusivity decreased with increasing vesicle complexity on all substrates. It was concluded that ∼20% of the pure POPC vesicles remained unruptured on the composite thin film whereas a defect-free supported lipid bilayer was confirmed on the mesoporous thin film. The mesoporous silica thin film was the superior substrate for bilayer formation, and we managed to form bilayers derived from native membrane vesicles with an even higher lipid diffusivity compared to conventional glass substrates. These results represent a significant advance in the development of bioelectronic sensors, providing a new tool for researchers to better investigate how ion channels and other components of the native cell membrane function.

## Supporting Information Available

AFM micrograph showing the surface topography of a CTF3 composite thin film, SEM micrographs of a defect in PEDOT:PSS/silica composite substrate (CTF4), and QCM-D frequency and dissipation showing deposition of POPC vesicles and DPPC vesicles on a CTF3-coated sensor (PDF)
