# Supplementary material for: Formation of Supported Lipid Bilayers Derived from Vesicles of Various Compositional Complexity on Conducting Polymer/Silica Substrates
Source: Langmuir. 2021 Apr 30;37(18):5494–505. doi: 10.1021/acs.langmuir.1c00175 (PMC8280725; doi:10.1021/acs.langmuir.1c00175)
Supplement: Supplementary file 1 — la1c00175_si_001.pdf [file la1c00175_si_001.pdf]

SUPPORTING INFORMATION

Formation of supported lipid bilayers derived from vesicles of various compositional complexity on conducting polymer/silica substrates

Hanna Ulmefors[a], Josefin Nissa[b], Hudson Pace[a], Olov Wahlsten[a], Anders Gunnarsson[c], Daniel T. Simon[b], Magnus Berggren[b], and Fredrik Höök[a]*

[a] Division of Nano and Biological Physics, Department of Physics, Chalmers University of Technology, 412 96 Gothenburg, Sweden.

[b] Laboratory of Organic Electronics, Department of Science and Technology, Linköping University, 601 74 Norrköping, Sweden.

[c] Discovery Sciences, BioPharmaceuticals R&D, AstraZeneca, Pepparedsleden 1, 431 83 Mölndal, Sweden.

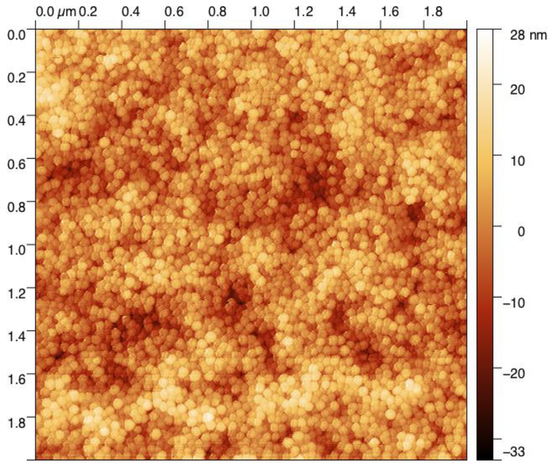

Figure S1. AFM micrograph showing the surface topography of a CTF3 composite thin film.

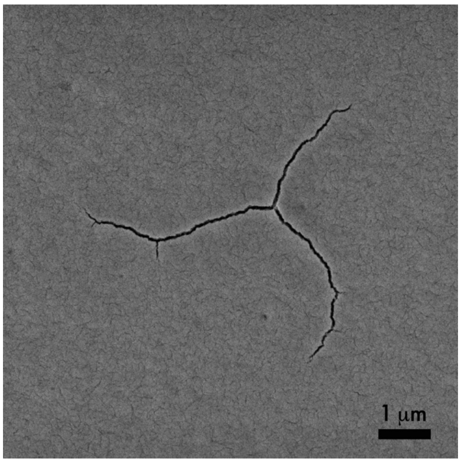

Figure S2. SEM micrographs of a defect in PEDOT:PSS/silica composite substrate (CTF4).

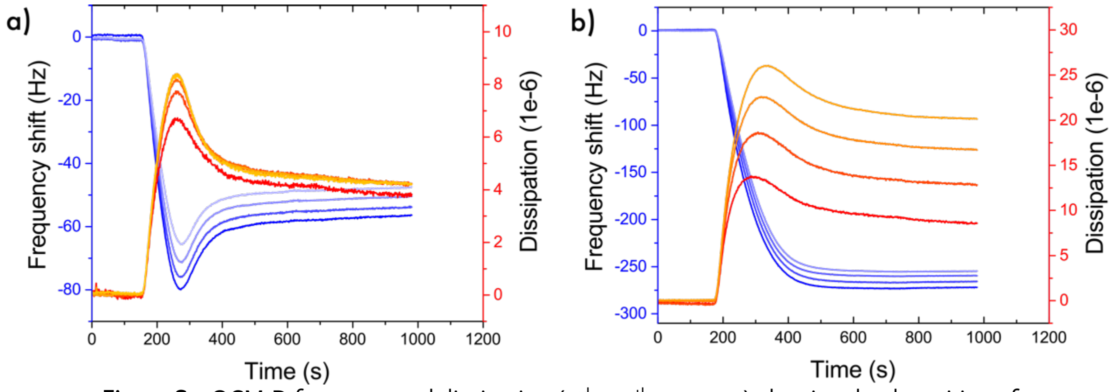

Figure S3. QCM-D frequency and dissipation (3rd to 9th overtone) showing the deposition of (a) POPC vesicles and (b) DPPC vesicles on a CTF3-coated sensor.
